# High Human Herpesvirus 8 (HHV-8) Prevalence, Clinical Correlates and High Incidence among Recently HIV-1-Infected Subjects in Sao Paulo, Brazil

**DOI:** 10.1371/journal.pone.0005613

**Published:** 2009-05-19

**Authors:** Mariana Dias Batista, Suzete Ferreira, Mariana M. Sauer, Helena Tomiyama, Maria Teresa Maidana Giret, Cláudio S. Pannuti, Ricardo S. Diaz, Ester C. Sabino, Esper G. Kallas

**Affiliations:** 1 Infectious Diseases Division, Federal University of São Paulo, São Paulo, Brazil; 2 Department of Dermatology, Federal University of São Paulo, São Paulo, Brazil; 3 Fundação Pró-Sangue, Hemocentro, São Paulo, Brazil; 4 Instituto de Medicina Tropical, University of São Paulo, São Paulo, Brazil; 5 Division of Clinical Immunology and Allergy, University of São Paulo, São Paulo, Brazil; Karolinska Institutet, Institution for Laboratory Medicine, Sweden

## Abstract

**Background:**

Human herpesvirus 8 (HHV-8) is the etiological agent for Kaposi Sarcoma, which occurs especially in HIV-infected subjects. HHV-8 infection and its clinical correlates have not been well characterized in recently HIV-1-infected subjects, especially men who have sex with men (MSM).

**Methodology/ Principal Findings:**

We assessed the HHV-8 seroprevalence, clinical correlates, and incidence after one year of follow-up in a cohort of 228 recently HIV-1-infected individuals, of whom 83.6% were MSM, using indirect immunofluorescence assay. The prevalence of HHV-8 infection at the time of cohort enrollment was 25.9% (59/228). In the univariate model, there were significant associations with male gender, black ethnicity, MSM practice, and previous hepatitis B virus and syphilis infections. In the multivariate model we could still demonstrate association with MSM, hepatitis B, and black ethnicity. No differences in mean CD4+ cell counts or HIV viral load according to HHV-8 status were found. In terms of incidence, there were 23/127 (18.1%) seroconversions in the cohort after 1 year.

**Conclusions:**

HHV-8 is highly prevalent among recently HIV-1-infected subjects. Correlations with other sexually transmitted infections suggest common transmission routes.

## Introduction

Human herpesvirus-8 (HHV-8) infection is not always associated with clinical manifestations [1]. Nonetheless, when these manifestations do occur, they can have a profound impact over quality of life [2]. Kaposi's sarcoma (KS) and other consequences of HHV-8 are much more likely to arise in immunosuppressed subjects, especially those HIV-infected. Therefore, studies of prevalence of HHV-8 among HIV-infected patients are of prime importance, as they can help estimate the risks of future co-infection-derived complications [3].

HIV affects HHV-8 through different mechanisms. It is debatable whether HIV Tat [4], inflammatory cytokines released during HIV infection [5], or immunosuppression itself are the main co-factors for the development of KS, but HIV has an unquestionable predisposing effect for the conversion from asymptomatic HHV-8 infection into clinical manifestations. Besides, AIDS-KS is more aggressive and resistant to treatment than other forms of KS [6]. HIV Tat activates lytic cycle replication of HHV-8, via JAK/STAT signaling [7], or by induction of HHV-8 Rta, a product of HHV-8 ORF 50 gene that controls the transition from latency to lytic replication [8].

Co-infections also have several effects on the course and progression of HIV. In this regard, the effects of HHV-8 infection over HIV natural history are complex and still not entirely elucidated [9]. Certain specific HHV-8 antigens such as LANA (latency-associated nuclear antigen) can activate HIV [10], and ORF 50, a lytic cycle gene, interacts with HIV Tat leading to increased cell susceptibility to HIV infection [11], [12]. HHV-8 stimulates HIV replication in acutely infected cells as well as reactivation in chronically infected cells [9].

Lastly, the order and timing in which these two infections occur can have prognostic implications. KS incidence is increased in people who seroconvert to HHV-8 after HIV, with hazard ratios of 2.55 [13] to 5.04 [3] and an additional risk of 1.6 in relation to HIV-infected persons who were previously infected by HHV-8 [3].

Little is known about the prevalence and clinical correlates of HHV-8 infection among recently HIV-infected individuals. We studied these characteristics among 228 recently HIV-infected individuals recruited in Sao Paulo, Brazil. In addition, we investigated the impact of HHV-8 co-infection over CD4+ T cell count and HIV-viral load. Finally, we examined the incidence of new HHV-8 seroconversions in this cohort after 1-year of follow-up.

## Methods

### Ethics Statement

This research obtained approval by the Ethics Committee and the Institutional Review Board of the Federal University of Sao Paulo and patients provided informed consent.

### Cohort description and laboratory measures

This study was performed in a cohort investigation that started recruiting recently HIV-infected people in 2002 in Sao Paulo, Brazil, aiming at the identification of host factors that contribute to progression to immunodeficiency [14], [15]. Recent HIV infection was determined by the Serologic Testing Algorithm for Recent HIV Seroconversion (STARHS), and individuals were included in the study when they had a negative desensitized ELISA HIV-test, that could indicate an incomplete antibody response as a consequence of recent HIV infection [15]. There were 237 volunteers initially included in the cohort, but 9 were excluded due to the presence of AIDS-defining conditions, representing false-positive STARHS indication of recent infection. As a result, 228 volunteers were prospectively followed in the cohort. Individuals were followed until the start of treatment, which happened when the CD4+ T cell count dropped below 300 cells/µl or AIDS-defining conditions developed.

Data on gender, age, ethnicity, mode of transmission, and presence of symptoms were collected. We examined CD4+ and CD8+ T cell counts and plasma HIV-1 RNA copies/ml at the initial and subsequent visits. CD4+ and CD8+ T cell counts were performed using a lymphocyte marking technique with anti- CD3, CD4 and CD8 conjugated monoclonal antibodies (Kit TriTest, BD Biosciences, San Diego, California, USA). The plasma RNA measurements were performed using a Amplicor HIV-1 Monitor test, version 1.5 (Roche Diagnostics, Indianapolis, IN, USA) until January 2007, and was then subsequently replaced by the bDNA (branched DNA) (Versant® - bDNA HIV-1 RNA 3.0 ASSAY, Bayer Health Care LLC Tarrytown, NY). All individuals in the cohort were tested for herpes simplex types 1 and 2, hepatitis B, C and G by indirect ELISA (GBC ELISA provided by Dietmar Zdunek, Roche Diagnostics, Germany; HSV-2 ELISA Diasorin, Saluggia, Italy), syphilis (MHA-Tp and FTA-abs), CMV, EBV, toxoplasmosis (Diasorin, Saluggia, Italy). HHV-8 serology was performed retrospectively, with samples collected in the first visit and after 1 year of follow-up. Table 1 summarizes cohort epidemiological and demographic data.

**Table 1:** Demographic and serologic characteristics of cohort at initial visit.

Demographics	-	Number	%
Gender	Male	207	90.8
	Female	21	9.2
Ethnicity	White	129	58.4
	Black	17	7.7
	Mixed	44	19.9
	Other	31	14
Exposure	MSM	188	83.6
	Heterosexual	37	16.4
Co-infections	HSV-2	89/145	61.4
	Anti-HBc	80/203	39.6
	Syphilis	40/203	19.8
**Variable**	-	**Median**	**IQR 25–75%**
CD4+ T cell count (cells/µl)	-	529	403–709
Plasma HIV viral load (log_10_ copies/ml)	-	4.26	3.59–4.81
Time to start on ART (days)	-	412	153–694

Antibodies to latent and lytic HHV-8 antigens were detected through an indirect immunofluorescence assay (IFA) based on the BCBL-1 cell line [16], [17]. Cells were grown in RPMI 1640 (Gibco BRL) supplemented with 10% fetal calf serum, antibiotics (penicillin and streptomycin), and amphotericin B. Cell cultures were then washed three times with phosphate buffered saline (PBS), resuspended in PBS and 10 µL of the suspension was smeared onto slides, with a concentration of 10×10^6^ cells/ml. The slides were air-dried and fixed for subsequent incubation for 30 minutes at 37°C with the test serum diluted at 1∶80 and a goat anti-human antibody fluorescein isothiocyanate-conjugate (Sigma 1∶100 in PBS/ Evans blue milk 0.01 mg/ml); the slides were then washed and dried. Punctuate nuclear staining was considered positive for antibodies against LANA in untreated cells. For induction of lytic replication, cells were treated with tetradecanoyl-phorbol ester acetate. Whole cell fluorescence in more than 20% of cells was considered positive for lytic antigens. Two researchers read the slides independently, and indeterminate results were repeated twice.

### Statistical Analysis

Statistical analyses were performed using SPSS 16 software (SPSS Inc., Chicago, Illinois, USA), with a minimum significance level of p = 0.05. Initially, we performed a descriptive analysis of demographic and laboratory results. Differences between groups by HHV-8 status were analyzed using Chi-Square or Fisher's Exact Test. Multivariate analyses were performed by logistic regression, with inclusion of variables with p≤0.05, and analysis by Hosmer and Lemeshow Test. We used the Mann-Whitney U-Test to compare differences in continuous variables. Besides, CD4+ T cell count and HIV viral load were also analyzed as categorical variables using the median values to define groups. The survival analysis was performed through a Kaplan-Meier curve with CD4+ T cell count ≤350 as the main outcome. We used the log-rank test to compare differences in the Kaplan Meier survival functions according to HHV-8 status.

## Results

The overall prevalence of antibodies against HHV-8 in the cohort was 25.9% (59/228). Of those 59 seropositives, 12 (20.3%) subjects were positive for latent and lytic antibodies concomitantly, 14 (23.7%) positive for LANA antibodies only, and 33 (55.9%) for lytic antibodies only. In the univariate analysis, described in Table 2, we were able to find correlations of HHV-8 infection with male gender (27.5% male HHV-8+ *vs*. 9.5% female HHV-8+, p = 0.05), MSM practice (28.7% MSM HHV-8+ *vs*. 10.8% heterosexuals HHV-8+, p = 0.023), previous hepatitis B (anti-hepatitis B core antibody) (19.7% anti-HBc-/HHV-8+ *vs*. 36.3% anti HBc+/HHV-8+, p = 0.009), syphilis infection (MHA-Tp and FTA-ABS) (23.5% syphilis-/HHV-8+ *vs*. 40% syphilis+/HHV-8+, p = 0.034), and black ethnicity (52.9% black/HHV-8+ *vs*. 29.5% mixed/HHV-8+ *vs*. 19.4% white/HHV-8+, p = 0.021).

**Table 2:** Correlates of HHV-8 infection in study cohort.

Variable	-	Univariate			Multivariate		
		OR*	95% CI**	p	OR*	95% CI**	p
Ethnicity	White	1.0	-	-	-	-	-
	Black	4.680	(1.642, 13.343)	0.005	9.068	(2.478, 33.176)	0.018
	Mixed	1.745	(0.799, 3.810)	0.160	1.556	(0.657, 3.684)	0.351
	Other	1.447	(0.579, 3.614)	0.427	1.672	(0.604, 4.625)	0.361
Hepatitis B (anti-HBc seropositivity) (N/P)	-	2.322	(1.227, 4.395)	0.009	2.230	(1.108, 4.488)	0.039
Syphilis (MHA-Tp) (N/P)	-	2.175	(1.049, 4.512)	0.034	1.519	(0.662, 3.485)	0.324
HSV-2 (N/P)	-	1.034	(0.494, 2.165)	0.929	-	-	-
Exposure MSM/hetero	-	0.301	(0.102, 0.890)	0.023	0.176	(0.041, 0.079)	0.023
Gender M/F	-	0.277	(0.063, 1.227)	0.054	3.841	(0.256, 57.519)	0.329

* OR: odds ratio.

** CI: confidence interval.

**Figure 1:**
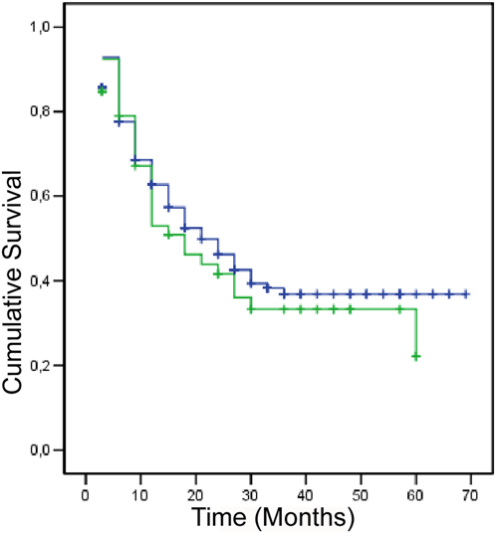
Kaplan-Meier survival curves with CD4+ T cell count ≤350 as the outcome. Legends: green line: HHV-8 negative; blue line: HHV-8 positive green; +: HHV-8 negative censored blue; +: HHV-8 positive censored.

We then compared the median age using the Mann-Whitney test and could not detect differences between the HHV-8 seronegative and seropositive groups (median = 31 in both groups, p = 0.836), nor did we find differences among the median CD4+ T cell counts and viral load values between the two groups in any of the follow-up visits. Antiretroviral treatment was not initiated more frequently in relation to HHV-8 status (24.9% treatment negative/HHV-8+ *vs*. 29.1% treatment+/HHV-8+, p = 0.3), and the median time until the start of antiretroviral treatment did not vary between the two groups (median time to treatment in HHV-8 negative = 421 days *vs*. 405 days in HHV-8+, p = 0.9). In a Kaplan-Meier survival analysis comparing HHV-8 positive and negative individuals with CD4+ T cell count ≤350 cells/mm^3^ as main outcome, there was no difference between groups by the log-rank test (Figure 1). In the multivariate analysis, we could still demonstrate association between HHV-8 status and MSM exposure, hepatitis B and black ethnicity (Table 2).

We repeated the HHV-8 serology after 1-year follow-up in 127 of the 169 initially HHV-8-negative individuals. There were 23/127 (18.1%) seroconversions in the cohort after one year. The 23 subjects who seroconverted after 1-year did not differ from the 59 HHV-8+ at the initial visit. In relation to the subjects who remained negative after one year of follow-up, the only statistically significant difference was observed in the time until start of antiretroviral treatment (median time to treatment in HHV-8 negative subjects 245 *vs*. 573 days in HHV-8+, p = 0.045). However, due to the fact that only 35 individuals initiated treatment among the 127 tested after one year of follow-up, this finding was not taken into further consideration.

## Discussion

In this work, we found a significant proportion of recently HIV-infected individuals who are seropositive for HHV-8 (25.9% overall; 28.7% among MSM), which was consistent with reported rates in HIV-1-infected subjects in Brazil, ranging from 14.6 to 18.7%, although higher among MSM (30.4 to 32.4%) [18], [19], [20]. An also high prevalence (32%) has been found in a group of recently HIV-1-infected military members in the US [21]. Of notice, we observed a correlation between HHV-8 and markers of sexual activity, such as hepatitis B and syphilis. This provides additional evidence for the hypothesis that risk behaviors associated with HIV-1 and other sexually transmitted infections (STIs) can also increase the probability for HHV-8 acquisition [21]. Other studies have shown an association between HHV-8 and STIs, especially among MSM [22], [23], [24]. HHV-8 acquisition has been associated with multiple sex partners in both MSM and heterosexuals, and practices involving saliva are thought to increase transmission, accounting for differences in incidence of HHV-8 and HIV [25].

In addition, we were able to detect an association between black ethnicity and HHV-8 in this cohort of recently HIV-infected individuals. In fact, previous reports have described such association between black ethnicity and HHV-8 in women [26], [27]. Although one study has found an association between black ethnicity and protection against HHV-8 infection in men, this was only observed among heterosexuals [21], different from the present cohort, predominantly constituted by MSM. The explanations for the difference in association between black ethnicity and HHV-8 according to sexual orientation are unclear and could again involve specific sexual practices [25].

The finding of 23/127 seroconversions after one year of follow-up could indicate ongoing exposure of individuals after HIV infection. HIV-infected subjects are at higher risk for HHV-8 seroconversion, and incident HHV-8 infection has been associated with older age and multiple sexual partners, as well as orogenital sexual practice and recreational drug use [28]. Incident HSV-2 infection has been previously assessed in 47 individuals from the cohort, with a total of 10 (21.2%) seroconversions [29]. The presence of incident co-infections supports the fact that HSV-2 and HHV-8 share common transmission routes in HIV-infected persons. Besides, individuals who seroconverted to HHV-8 after HIV are at a greater risk of subsequent development of KS [3], [13].

Conversely, the lack of short term repercussion of HHV-8 infection over CD4+ T cell counts and plasma HIV viral load during the observed follow-up period is in accordance with previous evidence that HHV-8 has little influence on the progression of HIV in initially asymptomatic individuals [30]. Indeed, we found that HHV-8 serostatus was not associated with the need for antiretroviral treatment, nor with a shorter time until the initiation of antiretroviral treatment.

HHV-8 serology was performed by immunofluorescence assays against latent and lytic HHV-8 antigens. The association of assays against latent and lytic antigens is prefered for asymptomatic populations [31]. Immunofluorescence assays for latent phase antibodies can have sensitivities ranging from 52 to 93%, yet these values can be affected by low CD4+ T cell counts, especially under 100 cells/ml [32]. In this regard, most individuals in this cohort had higher CD4+ T cell counts, as a result of their recent HIV infection. The association of immunofluorescence for lytic phase antibodies helped diminish this limitation. Although K8.1 EIA would have further increased sensitivity, it could also decrease specificity [33]. To add evidence to the aforementioned activating effect of HIV on HHV-8 replication [4], [5], we found that 45/59 (76.3%) of HHV-8-positive individuals had lytic phase antibodies.

In conclusion, HHV-8 infection is highly prevalent among recently HIV-infected individuals. Significant associations with sexually transmitted infections such as hepatitis B and syphilis add evidence to the theory of a common transmission route. The finding of a high incidence rate within the first year of follow-up of this cohort points to an ongoing exposure behavior after HIV acquisition. Safer sexual practices must be recommended to decrease risks associated with co-infection.

## References

[1] Casper C, Krantz E, Selke S, Kuntz SR, Wang J (2007). Frequent and asymptomatic oropharyngeal shedding of human herpesvirus 8 among immunocompetent men.. J Infect Dis.

[2] Harris AH, Osborne RH, Streeton CL, McNeil H (2002). Quality of life and Kaposi sarcoma: using preference techniques to value the health gains from treatment.. Support Care Cancer.

[3] Renwick N, Halaby T, Weverling GJ, Dukers NH, Simpson GR (1998). Seroconversion for human herpesvirus 8 during HIV infection is highly predictive of Kaposi's sarcoma.. AIDS.

[4] Ensoli B, Barillari G, Salahuddin SZ, Gallo RC, Wong-Staal F (1990). Tat protein of HIV-1 stimulates growth of cells derived from Kaposi's sarcoma lesions of AIDS patients.. Nature.

[5] Mercader M, Taddeo B, Panella JR, Chandran B, Nickoloff BJ (2000). Induction of HHV-8 lytic cycle replication by inflammatory cytokines produced by HIV-1-infected T cells.. Am J Pathol.

[6] Strathdee SA, Veugelers PJ, Moore PS (1996). The epidemiology of HIV-associated Kaposi's sarcoma: the unraveling mystery.. AIDS.

[7] Zeng Y, Zhang X, Huang Z, Cheng L, Yao S (2007). Intracellular Tat of human immunodeficiency virus type 1 activates lytic cycle replication of Kaposi's sarcoma-associated herpesvirus: role of JAK/STAT signaling.. J Virol.

[8] Varthakavi V, Smith RM, Deng H, Sun R, Spearman P (2002). Human immunodeficiency virus type-1 activates lytic cycle replication of Kaposi's sarcoma-associated herpesvirus through induction of KSHV Rta.. Virology.

[9] Caselli E, Galvan M, Cassai E, Caruso A, Sighinolfi L (2005). Human herpesvirus 8 enhances human immunodeficiency virus replication in acutely infected cells and induces reactivation in latently infected cells.. Blood.

[10] Hyun TS, Subramanian C, Cotter MA, Thomas RA, Robertson ES (2001). Latency-associated nuclear antigen encoded by Kaposi's sarcoma-associated herpesvirus interacts with Tat and activates the long terminal repeat of human immunodeficiency virus type 1 in human cells.. J Virol.

[11] Caselli E, Galvan M, Santoni F, Rotola A, Caruso A (2003). Human herpesvirus-8 (Kaposi's sarcoma-associated virus) ORF50 increases in vitro cell susceptibility to human immunodeficiency virus type 1 infection.. J Gen Virol.

[12] Caselli E, Menegazzi P, Bracci A, Galvan M, Cassai E (2001). Human herpesvirus-8 (Kaposi's sarcoma-associated herpesvirus) ORF50 interacts synergistically with the tat gene product in transactivating the human immunodeficiency virus type 1 LTR.. J Gen Virol.

[13] Jacobson LP, Jenkins FJ, Springer G, Munoz A, Shah KV (2000). Interaction of human immunodeficiency virus type 1 and human herpesvirus type 8 infections on the incidence of Kaposi's sarcoma.. J Infect Dis.

[14] Bassichetto KC, Bergamaschi DP, Oliveira SM, Deienno MC, Bortolato R (2008). Elevated risk for HIV-1 infection in adolescents and young adults in Sao Paulo, Brazil.. PLoS ONE.

[15] Kallas EG, Bassichetto KC, Oliveira SM, Goldenberg I, Bortoloto R (2004). Establishment of the serologic testing algorithm for recent human immunodeficiency virus (HIV) seroconversion (STARHS) strategy in the city of Sao Paulo, Brazil.. Braz J Infect Dis.

[16] Gao SJ, Kingsley L, Li M, Zheng W, Parravicini C (1996). KSHV antibodies among Americans, Italians and Ugandans with and without Kaposi's sarcoma.. Nat Med.

[17] Lennette ET, Blackbourn DJ, Levy JA (1996). Antibodies to human herpesvirus type 8 in the general population and in Kaposi's sarcoma patients.. Lancet.

[18] Caterino-de-Araujo A, Calabro ML, de los Santos-Fortuna E, Suleiman J, Chieco-Bianchi L (1999). Searching for human herpesvirus 8 antibodies in serum samples from patients infected with human immunodeficiency virus type 1 and blood donors from Sao Paulo, Brazil.. J Infect Dis.

[19] Zago A, Bourboulia D, Viana MC, Collandre H, Dietze R (2000). Seroprevalence of human herpesvirus 8 and its association with Kaposi sarcoma in Brazil.. Sex Transm Dis.

[20] Pierrotti LC, Etzel A, Sumita LM, Braga PE, Eluf-Neto J (2005). Human herpesvirus 8 (HHV-8) infection in HIV/AIDS patients from Santos, Brazil: seroprevalence and associated factors.. Sex Transm Dis.

[21] Crum NF, Wallace MR, Stephan K, Blazes DL, Aronson N (2003). Correlates of human herpesvirus-8 seropositivity among U.S. military members recently infected with human immunodeficiency virus.. Sex Transm Dis.

[22] Smith NA, Sabin CA, Gopal R, Bourboulia D, Labbet W (1999). Serologic evidence of human herpesvirus 8 transmission by homosexual but not heterosexual sex.. J Infect Dis.

[23] Casper C, Wald A, Pauk J, Tabet SR, Corey L (2002). Correlates of prevalent and incident Kaposi's sarcoma-associated herpesvirus infection in men who have sex with men.. J Infect Dis.

[24] Diamond C, Thiede H, Perdue T, MacKellar D, Valleroy LA (2001). Seroepidemiology of human herpesvirus 8 among young men who have sex with men.. Sex Transm Dis.

[25] Giuliani M, Cordiali-Fei P, Castilletti C, Di Carlo A, Palamara G (2007). Incidence of human herpesvirus 8 (HHV-8) infection among HIV-uninfected individuals at high risk for sexually transmitted infections.. BMC Infect Dis.

[26] Cannon MJ, Dollard SC, Smith DK, Klein RS, Schuman P (2001). Blood-borne and sexual transmission of human herpesvirus 8 in women with or at risk for human immunodeficiency virus infection.. N Engl J Med.

[27] Greenblatt RM, Jacobson LP, Levine AM, Melnick S, Anastos K (2001). Human herpesvirus 8 infection and Kaposi's sarcoma among human immunodeficiency virus-infected and -uninfected women.. J Infect Dis.

[28] Dukers NH, Renwick N, Prins M, Geskus RB, Schulz TF (2000). Risk factors for human herpesvirus 8 seropositivity and seroconversion in a cohort of homosexual men.. Am J Epidemiol.

[29] Barbour JD, Sauer MM, Sharp ER, Garrison KE, Long BR (2007). HIV-1/HSV-2 co-infected adults in early HIV-1 infection have elevated CD4+ T cell counts.. PLoS ONE.

[30] Ait-Arkoub Z, Robert-Visse C, Calvez V, Costagliola D, Autran B (2003). No influence of human herpesvirus 8 infection on the progression of HIV-1 infection in initially asymptomatic patients.. AIDS.

[31] Biggar RJ, Engels EA, Whitby D, Kedes DH, Goedert JJ (2003). Antibody reactivity to latent and lytic antigens to human herpesvirus-8 in longitudinally followed homosexual men.. J Infect Dis.

[32] de Souza VA, Pierrotti LC, Sumita LM, Freire WS, Segurado AA (2007). Seroreactivity to Kaposi's sarcoma-associated herpesvirus (human herpesvirus 8) latent nuclear antigen in AIDS-associated Kaposi's sarcoma patients depends on CD4+ T-cell count.. J Med Virol.

[33] Nascimento MC, de Souza VA, Sumita LM, Freire W, Munoz F (2007). Comparative study of Kaposi's sarcoma-associated herpesvirus serological assays using clinically and serologically defined reference standards and latent class analysis.. J Clin Microbiol.

